# Disulfiram/Cu Kills and Sensitizes *BRAF*-Mutant Thyroid Cancer Cells to *BRAF* Kinase Inhibitor by ROS-Dependently Relieving Feedback Activation of MAPK/ERK and PI3K/AKT Pathways

**DOI:** 10.3390/ijms24043418

**Published:** 2023-02-08

**Authors:** Jingyi Xie, Juan Liu, Man Zhao, Xinru Li, Yubo Wang, Yuelei Zhao, Hongxin Cao, Meiju Ji, Mingwei Chen, Peng Hou

**Affiliations:** 1 Key Laboratory for Tumor Precision Medicine of Shaanxi Province, The First Affiliated Hospital of Xi’an Jiaotong University, Xi’an 710061, China; 2 Department of Endocrinology, The First Affiliated Hospital of Xi’an Jiaotong University, Xi’an 710061, China; 3 Center for Translational Medicine, The First Affiliated Hospital of Xi’an Jiaotong University, Xi’an 710061, China; 4 Department of Respiratory and Critical Care Medicine, The First Affiliated Hospital of Xi’an Jiaotong University, Xi’an 710061, China

**Keywords:** thyroid cancer, disulfiram/Cu, *BRAF* kinase inhibitor, *HER3*, sensitizing effect

## Abstract

*BRAF^V600E^*, the most common genetic alteration, has become a major therapeutic target in thyroid cancer. Vemurafenib (PLX4032), a specific inhibitor of *BRAF^V600E^* kinase, exhibits antitumor activity in patients with *BRAF^V600E^*-mutated thyroid cancer. However, the clinical benefit of PLX4032 is often limited by short-term response and acquired resistance via heterogeneous feedback mechanisms. Disulfiram (DSF), an alcohol-aversion drug, shows potent antitumor efficacy in a copper (Cu)-dependent way. However, its antitumor activity in thyroid cancer and its effect on cellular response to *BRAF* kinase inhibitors remain unclear. Antitumor effects of DSF/Cu on *BRAF^V600E^*-mutated thyroid cancer cells and its effect on the response of these cells to *BRAF* kinase inhibitor PLX4032 were systematically assessed by a series of in vitro and in vivo functional experiments. The molecular mechanism underlying the sensitizing effect of DSF/Cu on PLX4032 was explored by Western blot and flow cytometry assays. DSF/Cu exhibited stronger inhibitory effects on the proliferation and colony formation of *BRAF^V600E^*-mutated thyroid cancer cells than DSF treatment alone. Further studies revealed that DSF/Cu killed thyroid cancer cells by ROS-dependent suppression of MAPK/ERK and PI3K/AKT signaling pathways. Our data also showed that DSF/Cu strikingly increased the response of *BRAF^V600E^*-mutated thyroid cancer cells to PLX4032. Mechanistically, DSF/Cu sensitizes *BRAF*-mutant thyroid cancer cells to PLX4032 by inhibiting *HER3* and AKT in an ROS-dependent way and subsequently relieving feedback activation of MAPK/ERK and PI3K/AKT pathways. This study not only implies potential clinical use of DSF/Cu in cancer therapy but also provides a new therapeutic strategy for *BRAF^V600E^*-mutated thyroid cancers.

## 1. Introduction

Thyroid cancer is the most common endocrine malignancy [1]. In recent decades, its incidence and mortality have rapidly increased worldwide [2,3,4]. Like other malignancies, the pathogenesis of thyroid cancer involves many genetic and epigenetic alterations [5,6]. Of them, the *BRAF^V600E^* mutation is the most representative genetic event. This mutation is the most frequently found in thyroid cancers, accounting for more than 90% of oncogenic *BRAF* mutations, and drives thyroid tumorigenesis and progression by activating the mitogen-activated protein kinase/extracellular signal-regulated protein kinase (MAPK/ERK) signaling pathway [7,8]. The presence of the *BRAF^V600E^* mutation is significantly associated with increased cancer-related mortality among patients with papillary thyroid cancer [9]. *BRAF* mutation-positive patients show higher recurrence rates than *BRAF* mutation-negative patients [10]. A high percentage of *BRAF^V600E^* alleles predicts a poorer outcome in papillary thyroid cancer (PTC) [11]. In recent years, *BRAF^V600E^* has gradually become a major therapeutic target for anaplastic thyroid cancer [12].

In recent years, the use of kinase inhibitors (KIs) has revolutionized the treatment of aggressive forms of endocrine cancer [13]. A variety of KIs have been tested in the last decade for the treatment of iodine-refractory differentiated thyroid cancer [14]. Vemurafenib (PLX4032), a small-molecule inhibitor of *BRAF^V600E^* kinase, has high specificity for the *BRAF^V600E^* oncoprotein and exhibits a potently inhibitory effect on the MAPK/ERK cascade in *BRAF*-mutant cancer cells, but not *BRAF* wild-type cells [15]. PLX4032 prolongs survival in patients with *BRAF*-mutant melanoma [16]. In a phase II trial by Brose et al. [17], although vemurafenib showed antitumor activity in patients with progressive *BRAF^V600E^*-mutant PTC refractory to radioactive iodine with a best overall response of 38.5%, there were still many patients who were not in remission. It has shown limited benefit in patients with thyroid and colorectal cancers [16,18,19]. The major cause is that PLX4032 releases the transcription repressor CTBP from the *HER3* promoter and promotes the transcription of the latter by suppressing ERK phosphorylation. Autocrine-secreted NRG1 then binds to *HER3* to trigger *HER3*/HER2 heterodimerization and receptor phosphorylation, thereby activating their downstream MAPK/ERK and PI3K/AKT pathways and eventually leading to the resistance to PLX4032 [16]. Another challenge with *BRAF^V600E^* kinase inhibitor monotherapy is the emergence of squamous cell carcinoma, which was found by Brose et al. [17]. There is also evidence indicating that hyperproliferative cutaneous events can be caused by hyperactive MAPK signaling [20,21,22]. Hence, it is pressing to develop a new strategy to improve the response of *BRAF^V600E^*-mutated thyroid cancer to PLX4032.

Disulfiram (tetraethylthiuram disulfide, DSF), a drug that has been widely used for decades as a treatment for chronic alcoholism, has safety and well-established pharmacokinetics at the dosage recommended by the US Food and Drug Administration (FDA) [23]. It is believed to be a specific and irreversible inhibitor of aldehyde dehydrogenase (ALDH), a core enzyme involved in the ethanol metabolism process. DSF causes the accumulation of acetaldehyde by inhibiting ALDH after ethanol absorption, thereby inducing an obviously unpleasant DSF-like reaction characterized by vasodilation, tachycardia and tachypnea with subsequent hypotension, encephalalgia, nausea and vomiting [24]. In recent years, DSF showed antitumor activity in preclinical models [25,26,27]. Although the mechanism of its antitumor activity remains unclear, there is evidence showing that DSF inhibits proteasome activity and NF-ΚB translocation [28] and increases cellular ROS levels [29]. Several studies have shown that DSF chelates bivalent metals and forms complexes with copper (Cu), enhancing its antitumor activity [26,30,31]. Nevertheless, the antitumor role of DSF/Cu in thyroid cancer and its effect on the response of *BRAF^V600E^*-mutated thyroid cancer cells to PLX4032 have not yet been investigated.

In this study, we demonstrate that DSF/Cu can kill *BRAF^V600E^*-mutated thyroid cancer cells and improves their cellular response to *BRAF* kinase inhibitor by relieving feedback activation of MAPK/ERK and PI3K/AKT pathways in an ROS-dependent manner.

## 2. Results

### 2.1. Copper (Cu) Improves Antitumor Efficacy of Disulfiram in BRAF^V600E^-Mutated Thyroid Cancer Cells

**Figure 1:**
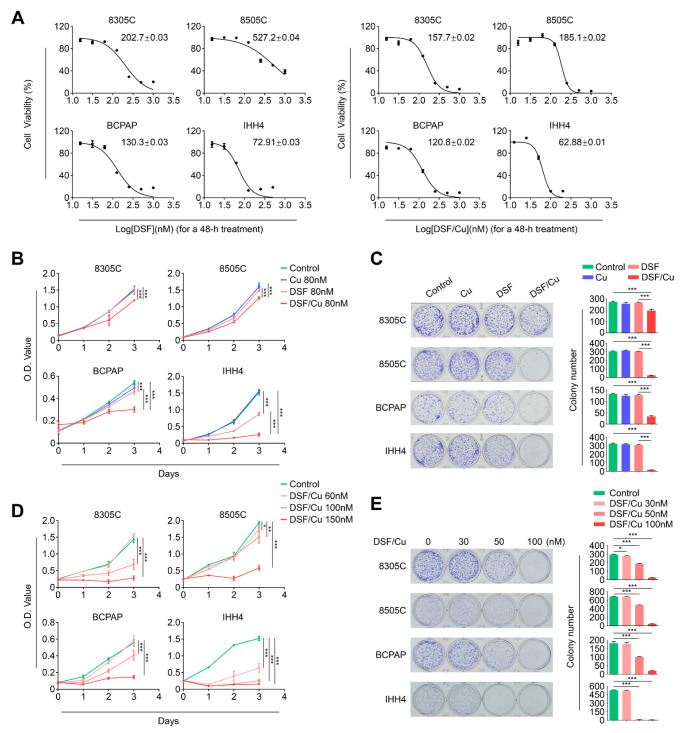
Copper (Cu) enhances the antitumor activity of disulfiram in *BRAF^V600E^*-mutated thyroid cancer cells. (**A**) *BRAF^V600E^*-mutated thyroid cancer cell lines 8305C, 8505C, BCPAP and IHH4 were treated with different concentrations of DSF or DSF/Cu (at 1:1 molar ratio) for 48 h. Cell viability was evaluated by MTT assay, and half-maximal inhibitory concentration (IC50) values were then calculated using the Reed–Muench method. (**B**) The above cells were treated with 80 nM DSF and 80 nM CuCl_2_, individually or in combination, for the indicated times. MTT assay was then performed to assess the proliferation of these cells. (**C**) Cells were treated with the indicated concentrations of DSF and CuCl_2_, individually or in combination (8305C: 80 nM DSF and/or 80 nM CuCl_2_; 8505C: 120 nM DSF and/or 120 nM CuCl_2_; BCPAP: 100 nM DSF and/or 100 nM CuCl_2_; IHH4: 50 nM DSF and/or 50 nM CuCl_2_) for 9 days, and then stained with crystal violet. The left panels show the representative images of colony formation. Quantitative analysis of colony numbers are presented in the right panels. (**D**) Cells were treated with different doses of DSF/Cu for the indicated times. MTT assay was then performed to evaluate their effects on cell viability. (**E**) Colony formation assay was performed when these cells were treated with DSF/Cu in dose-dependent manner for 9 days (left panels). Quantitative analysis of colony numbers is presented in the right panels. Data presented as means ± SD. * *p* < 0.05; ** *p* < 0.01; *** *p* < 0.001.

To determine the growth response of *BRAF^V600E^*-mutated thyroid cancer cell lines 8305C, 8505C, BCPAP and IHH4 to DSF, we treated these cells with incremental doses of DSF for 48 h to calculate half-maximal inhibitory concentration (IC50) using MTT assays. We also treated these cells with the same doses of DSF in combination with Cu and calculated their IC50. The results showed that DSF combined with the same dose of Cu showed lower IC50 values than DSF treatment alone (Figure 1A). Next, we tested the time-dependent cellular response towards DSF and Cu treatment alone or in combination. The results showed that a combined treatment of DSF and Cu displayed a stronger inhibitory effect on cell proliferation than DSF or Cu treatment alone (Figure 1B). This was further supported by colony formation assay (Figure 1C). Additionally, our data showed that DSF/Cu significantly inhibited cell proliferation in both a dose- and time-dependent way (Figure 1D). Colony formation assay further supported this conclusion (Figure 1E). Our data, taken together, indicate that Cu improves antitumor efficacy of DSF in *BRAF^V600E^*-mutated thyroid cancer cells.

### 2.2. DSF/Cu Potently Kills BRAF^V600E^-Mutated Thyroid Cancer Cells by Increasing Cellular ROS Levels

**Figure 2:**
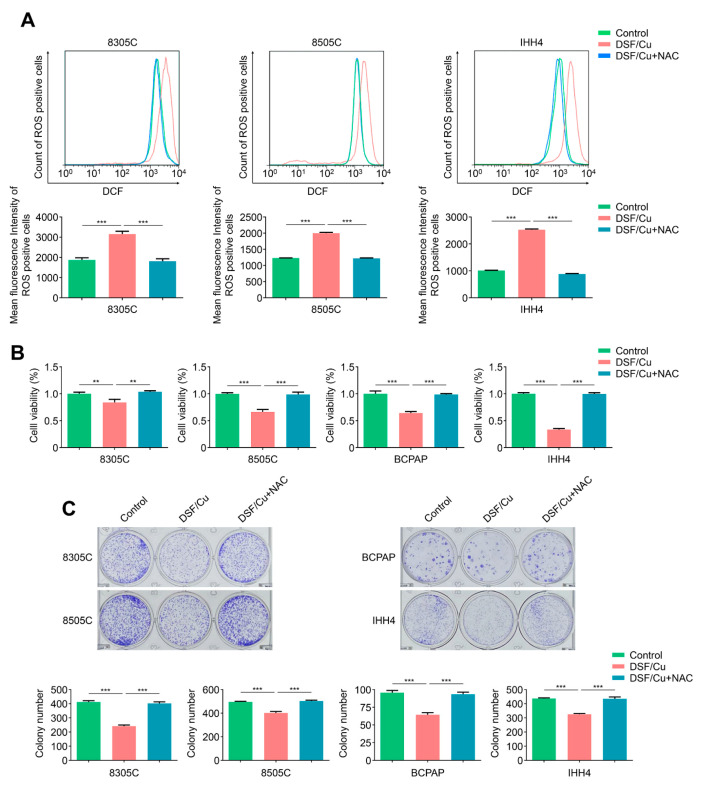
DSF/Cu kills thyroid cancer cells in an ROS-dependent way. (**A**) *BRAF^V600E^*-mutated thyroid cancer cell lines 8305C, 8505C and IHH4 were treated with 0.5 μM DSF/Cu alone or in combination with 2 mM NAC for 48 h, followed by a 1.5 h incubation with ROS-sensitive fluorescent dye DCF-DA. The ROS-positive cells were measured by flow cytometer (upper panels), and the mean fluorescence intensity of three independent experiments was then calculated by Student’s t test (lower panels). (**B**) The indicated cells were treated with 80 nM DSF/Cu alone or in combination with 2 mM NAC for 48 h, and cell viability was then measured by MTT assay. (**C**) Colony formation assay was performed when these cells were treated with DSF/Cu (8305C, 8505C and BCPAP: 50 nM, IHH4: 40 nM) alone or in combination with 2 mM NAC for 8–10 days. Data presented as means ± SD. ** *p* < 0.01; *** *p* < 0.001.

Reactive oxygen species (ROS) are oxygen-containing chemicals with reactive chemical properties produced by the respiratory chain reaction of mitochondria [32]. Although cancer cells have high ROS levels compare with normal tissues [33], there is evidence showing that elevated ROS levels can also deplete cellular antioxidative capacity, causing cancer cells to be unable to tolerate the ROS threshold and leading to cell death [34]. Next, we incubated 8305C, 8505C, BCPAP and IHH4 cells with DSF/Cu and measured cellular ROS levels. The results showed that DSF/Cu significantly elevated cellular ROS levels in comparison with the control, which was in line with previous reports indicating DSF/Cu potently kills cancer cells by substantially elevating endogenous ROS [29,30], and ROS scavenger NAC could effectively reverse this effect (Figure 2A and Figure S1). As expected, we found that DSF/Cu could significantly suppress cell proliferation and colony formation in 8305C, 8505C, BCPAP and IHH4 cells compared with the control, and these effects were completely reversed upon NAC treatment (Figure 2B,C). Altogether, the above results indicate that DSF/Cu kills *BRAF^V600E^*-mutated thyroid cancer cells by elevating endogenous ROS levels.

### 2.3. DSF/Cu Blocks MAPK/ERK and PI3K/AKT Signaling Pathways in Thyroid Cancer Cells in an ROS-Dependent Manner

**Figure 3:**
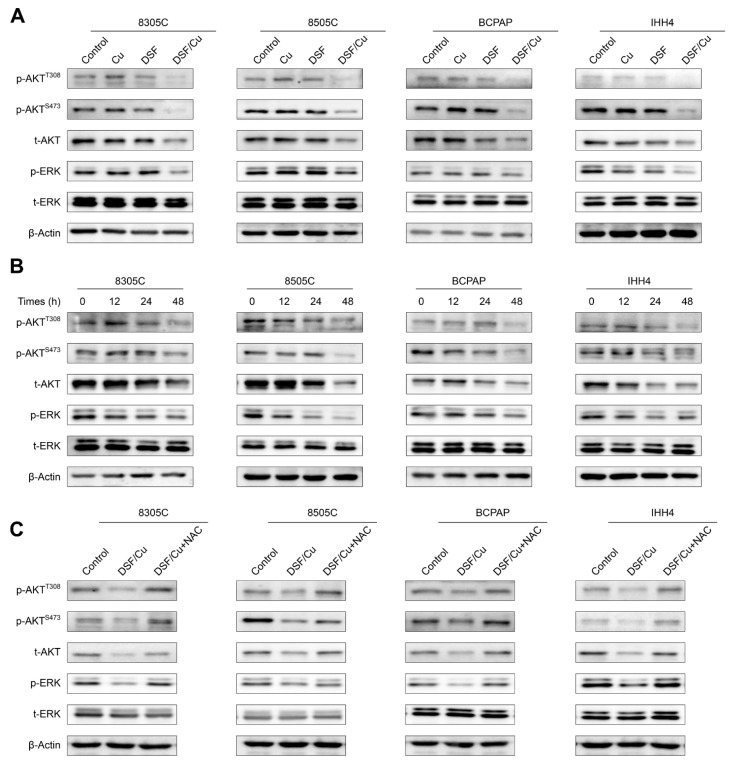
DSF/Cu inhibits the activities of MAPK/ERK and PI3K/AKT pathways via an ROS-dependent mechanism. (**A**) The indicated cells were treated with vehicle control, 0.5 μM DSF and/or 0.5 μM CuCl_2_ for 48 h, and cell lysates were then subjected to Western blot analysis to determine their effects on the levels of phosphorylated ERK (p-ERK), total ERK (t-ERK), phosphorylated AKT at Ser 473 (p-AKT^S473^), phosphorylated AKT at Thr308 (p-AKT^T308^) and total AKT (t-AKT). β-Actin was used as a loading control. (**B**) Western blot analysis of p-ERK, t-ERK, p-AKT^S473^, p-AKT^T308^ and t-AKT in indicated cells treated with vehicle control or 0.5 μM DSF/Cu for 0, 12 h, 24 h and 48 h, with β-Actin as a loading control. (**C**) Western blot analysis of p-ERK, t-ERK, p-AKT^S473^, p-AKT^T308^ and t-AKT in indicated cells treated with 0.5 μM DSF/Cu or in combination with 2 mM NAC. β-Actin was also used as a loading control.

Given the essential role of MAPK/ERK and PI3K/AKT pathways in thyroid tumorigenesis and progression [5], we next assessed the effect of DSF/Cu on the activities of these two pathways. First, we cultured 8305C, 8505C, BCPAP and IHH4 cells with DSF and Cu, individually or in combination. The results showed that DSF/Cu strongly suppressed the levels of phosphorylated AKT at Thr308 and Ser473, total AKT and phosphorylated ERK, while DSF or Cu treatment alone showed no effect on their levels (Figure 3A). Next, we treated the above cell lines with the combination of DSF and Cu for 0, 12 h, 24 h and 48 h, and expectedly found that DSF/Cu reduced the levels of phosphorylated AKT at Thr308 and Ser473, total AKT and phosphorylated ERK a time-dependent manner (Figure 3B). To further determine whether DSF/Cu-induced inhibition of these two pathways is mediated by ROS, we incubated 8505C, BCPAP, IHH4 and 8305C cells with DSF/Cu and ROS scavenger NAC, individually or in combination. The results showed that NAC could efficiently reverse the inhibitory effect of DSF/Cu on these two pathways (Figure 3C). The above results indicate that DSF/Cu suppresses the activities of MAPK/ERK and PI3K/AKT signaling pathway in thyroid cancer cells in an ROS-dependent way.

### 2.4. The Combination of DSF/Cu and PLX4032 Synergistically Kills BRAF^V600E^-Mutated Thyroid Cancer Cells

**Figure 4:**
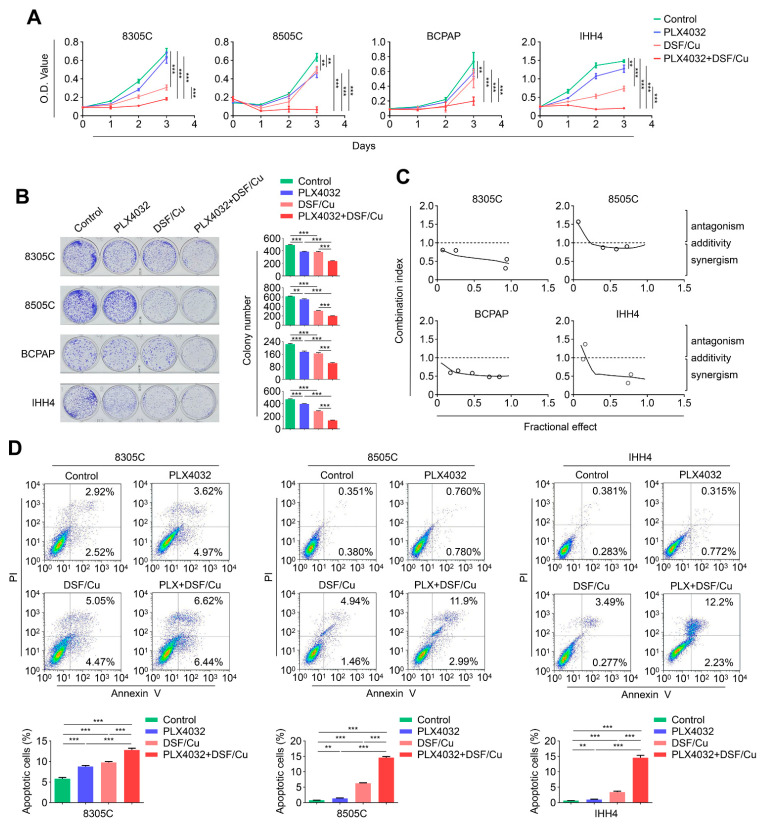
The combination of DSF/Cu and PLX4032 has a synergistic antitumor effect in *BRAF^V600E^*-mutated thyroid cancer cells. (**A**) The indicated cells were treated with 100 nM DSF/Cu (1:1) and 2 μM PLX4032, individually or in combination, for 48 h, and their effects on cell proliferation were then assessed by MTT assay. (**B**) The above cells were treated with 50 or 40 nM DSF/Cu (1:1) and 1 μM PLX4032, individually or in combination, for 8–10 days, and their effects on colony formation was further evaluated. The left panels show the representative images of colony formation. Quantitative analysis of colony numbers is presented in the right panels. (**C**) Dose–effect relationship between DSF/Cu and PLX4032 for antiproliferative effect was analyzed after 48 h of exposure in the indicated cells by the Chou–Talalay dose–effect method, and combination index (CI) values were calculated. CI < 1, CI = 1, and CI > 1 represent synergism, additivity and antagonism of these two agents, respectively. (**D**) *BRAF^V600E^*-mutated thyroid cancer cell lines 8305C, 8505C and IHH4 were treated with 0.5 μM DSF/Cu (1:1) and 2 μM PLX4032, individually or in combination, for 48 h, and their effects on cell apoptosis were analyzed by flow cytometry. Data presented as means ± SD. ** *p* < 0.01; *** *p* < 0.001.

To test the effect of DSF/Cu on cellular response of *BRAF* kinase inhibitor PLX4032, we first treated 8305C, 8505C, BCPAP and IHH4 cells with DSF/Cu and PLX4032, individually or in combination. The results indicated that a combined treatment of DSF/Cu and PLX4032 exhibited a stronger inhibitory action on cell proliferation than either DSF/Cu or PLX4032 monotherapy (Figure 4A). This was further supported by colony formation assays (Figure 4B). Next, we used Chou–Talalay method [35,36,37,38,39,40,41,42,43,44,45,46,47,48,49,50,51,52,53,54,55,56,57] to calculate combination index (CI) values in 8305C, 8505C, BCPAP and IHH4 cells. Expectedly, we observed a synergistic effect between DSF/Cu and PLX4032 in these four cell lines (Figure 4C). We also investigated the effect of DSF/Cu and PLX4032 monotherapy or in combination on cell apoptosis in 8505C, IHH4, 8305C and BCPAP cells. The results showed that a combined treatment induced more obvious cell apoptosis compared with either DSF/Cu or PLX4032 monotherapy (Figure 4D and S2). The above results, taken together, indicate that a combined treatment of DSF/Cu and PLX4032 exhibits a synergistic effect to kill *BRAF^V600E^*-mutated thyroid cancer cells.

### 2.5. DSF/Cu Improves the Response of BRAF^V600E^-Mutated Thyroid Cancer Cells to PLX4032 by Relieving Feedback Activation of MAPK/ERK and PI3K/AKT Signaling Pathways

**Figure 5:**
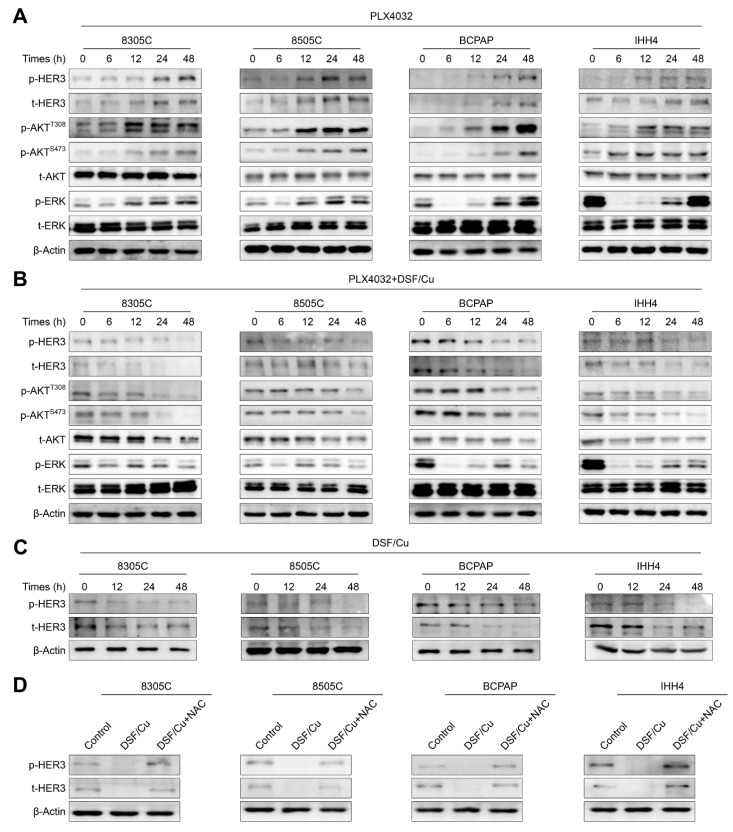
DSF/Cu relieves the rebound of MAPK/ERK and the activation of PI3K/AKT pathways caused by PLX4032. (**A**) The indicated cells were treated with 4 μM PLX4032 for 0 h, 6 h, 12 h, 24 h and 48 h, and Western blot analysis was then used to detect the levels of phosphorylated *HER3* (p-*HER3*), total *HER3* (t-*HER3*), p-AKT^S473^, p-AKT^T308^, t-AKT, p-ERK and t-ERK. (**B**) These cells were treated with the combination of 4 μM PLX4032 and 0.5 μM DSF/Cu, and Western blot analysis was similarly performed to detect the levels of the above molecules. (**C**) The same cell lines were treated with 0.5 μM DSF/Cu for 0 h, 12 h, 24 h and 48 h, and Western blot analysis was used to determine the levels of p-*HER3* and t-*HER3*. (**D**) Western blot analysis of p-*HER3* and t-*HER3* in the indicated cells treated with 0.5 μM DSF/Cu alone or in combination with 2 mM NAC. β-Actin was used as a loading control.

Previous studies have demonstrated that *BRAF^V600E^*-mutated thyroid cancer cells are usually insensitive to PLX4032. This is largely due to feedback upregulation of *HER3* expression, thereby activating its downstream MAPK/ERK and PI3K/AKT pathways [16]. Thus, we first treated 8305C, 8505C, BCPAP and IHH4 cells with PLX4032 for the indicated time points, and then assessed its effect on the activities of *HER3* signaling and its downstream MAPK/ERK and PI3K/AKT pathways. The results indicated that PLX4032 markedly down-regulated the level of phosphorylated ERK after 6 h treatment, while its level was gradually elevated after 12 h treatment (Figure 5A). Correspondingly, the levels of phosphorylated *HER3*, total *HER3* and phosphorylated AKT at Ser473 and Thr308 were also increased at this time point, which was consistent with a previous study [16].

Considering that there is a synergistic antitumor effect between DSF/Cu and PLX4032 in *BRAF^V600E^*-mutated thyroid cancer cells, we thus speculated that DSF/Cu may improve cellular response to PLX4032 by relieving feedback activation of *HER3* signaling and its downstream pathways. To verify this, we cultured 8305C, 8505C, BCPAP and IHH4 cells with DSF/Cu and PLX4032, individually or in combination, and expectedly found that a combined treatment clearly attenuated the rebound of phosphorylated ERK and decreased the levels of phosphorylated *HER3*, total *HER3*, phosphorylated AKT and total AKT compared with PLX4032 treatment alone (Figure 5B). In addition, our results indicated that DSF/Cu reduced the levels of phosphorylated *HER3* and total *HER3* in a time-dependent way (Figure 5C), and NAC supplement could effectively reverse this effect (Figure 5D). Altogether, the above findings indicate that DSF/Cu improves the response of *BRAF^V600E^*-mutated thyroid cancer cells to PLX4032 by relieving feedback activation of MAPK/ERK and PI3K/AKT signaling pathways.

### 2.6. DSF/Cu Potentiates the Antitumor Efficacy of PLX4032 in Nude Mice

**Figure 6:**
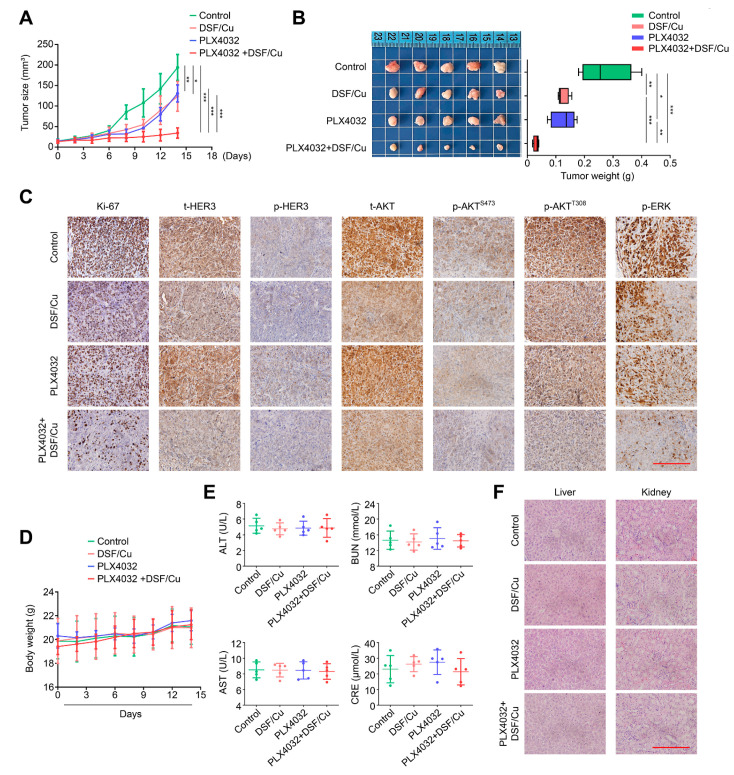
DSF/Cu exhibits a sensitizing effect on antitumor efficacy of PLX4032 in nude mice. A xenograft tumor model was established by subcutaneously injecting 8305C cells into the axilla of nude mice, and these mice were then randomly grouped and treated with DSF/Cu (DSF: 50 mg/kg; CuCl_2_: 0.15 mg/kg) and PLX4032 (50 mg/kg) alone or in combination, once a day. (**A**) Growth curves of xenograft tumors were drawn in each group. (**B**) Left and right panels show the pictures of dissected tumors and statistical results of tumor weight from the indicated groups. (**C**) IHC staining of Ki-67, t-*HER3*, p-*HER3*, t-AKT, p-AKT^S473^, p-AKT^T308^ and p-ERK in tumor tissues from the indicated group. Scale bar: 200 μm. (**D**) Growth cures of body weight were drawn in each group. (**E**) The levels of alanine transaminase (ALT), aspartate aminotransferase (AST), serum creatinine (CRE) and blood urea nitrogen (BUN) were measured by ELISA assay. (**F**) Representative H&E-stained slices of kidney and liver sections from the indicated mice. Scale bar: 200 μm. Data presented as means ± SD. * *p* < 0.05; ** *p* < 0.01; *** *p* < 0.001.

To further validate *in vivo* antitumoral activity of DSF/Cu, we first used the *BRAF^V600E^*-mutated thyroid cancer cell line 8305C to establish a nude mouse xenograft tumor model, and treated them with DSF/Cu and PLX4032, individually or in combination. The results indicated that a combined treatment of DSF/Cu and PLX4032 significantly retarded the growth of xenograft tumors (Figure 6A) and caused a tremendous reduction in tumor wight (Figure 6B) compared with the control and either DSF/Cu or PLX4032 monotherapy. The IHC assays also showed that a combined therapy of DSF/Cu and PLX4032 produced a remarkable decrease of Ki-67-positive cells in comparison with DSF/Cu or PLX4032 monotherapy (Figure 6C; Supplementary Figure S3). Similarly, combined treatment obviously decreased the levels of total *HER3*, phosphorylated *HER3*, total AKT, phosphorylated AKT and phosphorylated ERK compared with each monotherapy (Figure 6C). Importantly, we failed to find an obvious discrepancy in body weight (Figure 6D) among these four groups. There was likewise not any difference in the levels of glutamic pyruvic transaminase (GPT/ALT), glutamic oxaloacetic transaminase (GOT/AST), urea nitrogen (BUN) and creatinine (CRE) among them (Figure 6E), suggesting that the above treatments did not cause severe hepatorenal toxicity. This was also supported by H&E staining (Figure 6F). Our results, taken together, suggest that the combination of DSF/Cu and PLX4032 may be a safe and effective strategy for the treatment of *BRAF^V600E^*-mutated thyroid cancers.

### 2.7. DSF/Cu Alleviates the Feedback Activation of HER3 Signaling by PLX4032 in an ROS-Dependent Manner

**Figure 7:**
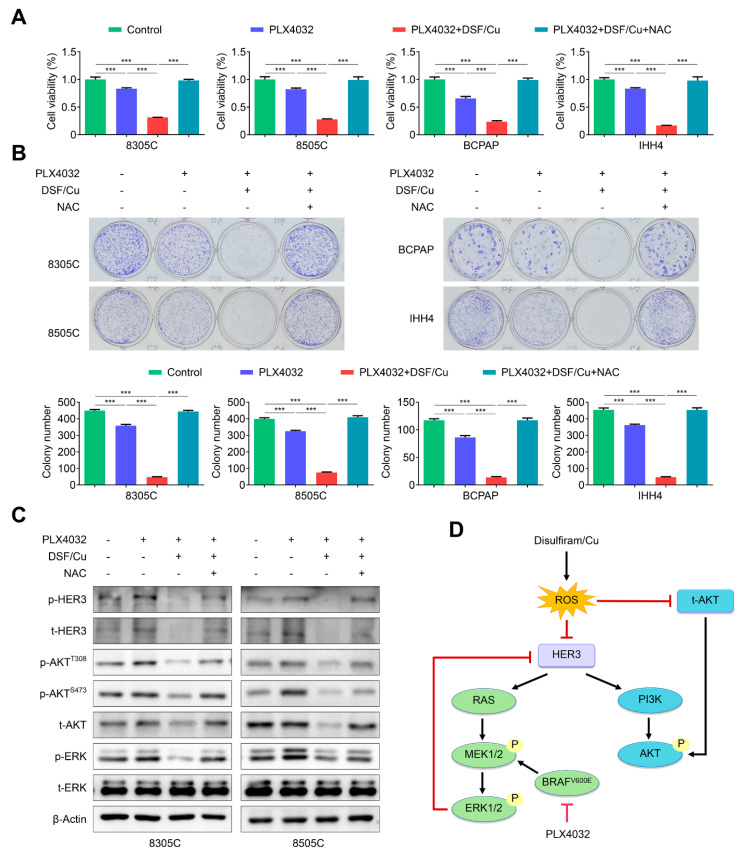
DSF/Cu improves antitumor effect of PLX4032 by ROS-dependently alleviating the feedback activation of *HER3* signaling. (**A**) The indicated cells were treated with vehicle control, 2 μM PLX4032, and 2 μM PLX4032 plus 100 nM DSF/Cu with or without 2 mM NAC for 48 h, and their effects on cell proliferation was determined by MTT assay. (**B**) Colony formation was evaluated in the above cells with the indicated treatments for 9 days. (**C**) Western blot analysis of t-*HER3*, p-*HER3*, t-AKT, p-AKT^S473^, p-AKT^T308^, t-ERK and p-ERK in the indicated cells with 4 μM PLX4032, and 4 μM PLX4032 plus 0.5 μM DSF/Cu with or without 2 mM NAC for 48 h. (**D**) A schematic model of DSF/Cu improving the response of *BRAF^V600E^*-mutated thyroid cancer cells to PLX4032. Data presented as means ± SD. *** *p* < 0.001.

As mentioned above, our data indicated that DSF/Cu ROS-dependently reduced the levels of total *HER3* and phosphorylated *HER3* and suppressed the activities of MAPK/ERK and PI3K/AKT pathways. Thus, we speculated that DSF/Cu enhanced the response of *BRAF^V600E^*-mutated thyroid cancer cells to PLX4032, probably by an ROS-dependent mechanism. To validate this, we first treated 8305C, 8505C, BCPAP and IHH4 cells with vehicle control, PLX4032, DSF/Cu + PLX4032 or DSF/Cu + PLX4032 + NAC, and tested their efficacy on cell viability and colony formation. Our data indicated that a combined treatment significantly suppressed cell viability and colony formation in comparison with PLX4032 treatment alone, and this effect was distinctly reversed by NAC (Figure 7A,B). Expectedly, our data also indicated that a combined therapy of DSF/Cu and PLX4032 potently attenuated the rebound of total *HER3*, phosphorylated *HER3*, phosphorylated AKT and phosphorylated ERK caused by PLX4032, while NAC supplement effectively reversed this effect (Figure 7C).

Summarizing the above-mentioned results, a schematic model was proposed to illustrate the mechanism of DSF/Cu improving antitumor efficacy of PLX4032 in *BRAF^V600E^*-mutated thyroid cancer cells (Figure 7D). Briefly, the treatment of *BRAF^V600E^*-mutated thyroid cancer cells with PLX4032 causes a feedback activation of MAPK/ERK and PI3K/AKT signaling through enhancing *HER3* transcription, leading to resistance to PLX4032. However, DSF/Cu relieves this feedback activation by ROS-dependently reducing the expression of *HER3* and AKT, thereby improving the response of *BRAF^V600E^*-mutated thyroid cancer cells to PLX4032.

## 3. Discussion

*BRAF^V600E^* mutation, the most common genetic alteration in thyroid cancer, has been recognized as a driver for the pathogenesis of most PTCs and some PTC-derived anaplastic thyroid cancers (ATCs) [5,8]. Direct association of *BRAF^V600E^* mutation with clinical progression, tumor recurrence and treatment failure of thyroid cancers has been demonstrated [8]. *BRAF* mutation has even been associated with PTC recurrence in patients with traditional low-risk clinicopathological factors [8]. Therefore, *BRAF* mutation has become a new prognostic biomarker and an attractive therapeutic target of ATC [7,38].

Specific *BRAF* kinase inhibitors have been widely developed in recent years. Since 2021, three *BRAF* inhibitors have been approved by the FDA, including vemurafenib, dabrafenib and encorafenib [39]. These *BRAF* kinase inhibitors specifically bind to the ATP-binding pocket of *BRAF* kinase and have a preference for *BRAF^V600E^* [22]. Dabrafenib is the first FDA-approved *BRAF* kinase inhibitor for the treatment of *BRAF^V600E^*-mutant ATC [40]. The phase II ROAR basket study confirm the substantial clinical benefit and manageable toxicity of dabrafenib plus trametinib in *BRAF^V600E^*-mutant ATC [41]. The first approved *BRAF* kinase inhibitor vemurafenib, as the first-line drug for *BRAF*-mutated metastatic melanoma, exhibited significantly clinical effects [42,43,44]. There was also a phase II trial confirming that vemurafenib exhibited antitumor activity in patients with progressive, *BRAF*-mutant PTC refractory to radioactive iodine [17]. After 6–7 months of treatment with *BRAF* kinase inhibitors, about half of the patients exhibited disease progression, finally leading to acquired resistance [22]. Unlike melanoma, due to the rebound of MAPK signaling [16,45,46], patients with *BRAF^V600E^*-mutated thyroid cancers exhibited few benefits from *BRAF* kinase inhibitors. Specifically, *BRAF* kinase inhibitors such as vemurafenib transiently repress ERK phosphorylation and derepresses *HER3* transcription. This will cause the rebound of phosphorylated ERK and the activation of AKT signaling, thereby attenuating their antitumor effects [16] and causing adverse events such as squamous cell carcinoma of the skin [17,20,21]. Thus, it is urgent to find strategies to solve the resistance of *BRAF* kinase inhibitors.

It has been demonstrated that the dual EGFR/HER-2 kinase inhibitor lapatinib sensitizes *BRAF*-mutant thyroid cancers to *BRAF* kinase inhibitors by blocking the rebound of MAPK/ERK pathway and the activation of the PI3K/AKT pathway [16]. There is also a study indicating that microphthalmia-associated transcription factor (MITF) inhibitor CH6868398 sensitized melanoma cells to *BRAF* kinase inhibitor [47]. Combined with Ref-1 inhibitor, it can also make *BRAF*-mutant thyroid cancers more sensitive to vemurafenib by inhibiting the MAPK/ERK pathway [48]. In addition, the combination of vemurafenib and tubulin inhibitor ABI-274 has been proved to overcome the resistance caused by vemurafenib in *BRAF*-mutant melanomas [49]. Although a large number of small-molecule drugs have been investigated to relive resistance in combination with *BRAF* kinase inhibitors, most of them are not FDA-approved, and their safety and efficacy need to be verified in further clinical trials.

Considering the high costs, long periods and high failure rate of developing new medicines, strategies to reduce costs, periods and improve success rates are thus urgently needed [50]. Drug repurposing is an effective strategy to solve some medical needs in cancer treatment by the use of FDA-approved drugs developed in other disease areas rather than by redeveloping new drugs [51]. This will save cost, time required, and risk. For example, disulfiram (DSF) has been FDA-approved for the treatment of alcohol abuse for more than 70 years [23]. It has been demonstrated to have many other effects beyond its original indication, such as the inhibitory effect on osteoclast differentiation and function [52], antiadipogenic effect, anti-inflammatory and antifibrotic effects in Graves’s orbitopathy [53], as well as antiproliferative effect on cancer cells [54]. There is growing evidence that DSF has great potential against various cancers, including melanoma [55], prostate cancer [25], breast cancer [26,31] and hepatocellular carcinoma [56]. Nevertheless, its role in thyroid cancer remains largely unclear.

Our present study showed that DSF could kill *BRAF^V600E^*-mutated thyroid cancer cells and copper (Cu) significantly enhanced this inhibitory effect, which was consistent with previous studies [26,31,55]. Hydrogen peroxide (H_2_O_2_) is an essential compound for the synthesis of thyroid hormone. It is an essential cofactor for thyroperoxidase, which catalyzes the final step of hormone production [57]. H_2_O_2_ produced within the thyrocyte by the DUOX and the NOX enzymes at higher concentrations than other human cells. H_2_O_2_ generation by DUOX at the apex of thyroid cells is the limiting factor in the oxidation of iodide and the synthesis of thyroid hormones [58]. The defect of such apparatus produces dramatic impairment of thyroid function, as demonstrated by the presence of congenital hypothyroidism in patients carrying mutations in the *DUOX2* gene [59]. There are studies showing that DSF/Cu induces cellular ROS production [30,55,60], as supported by our data that DSF/Cu suppressed the proliferation and colony formation of thyroid cancer cells by increasing cellular ROS levels, and NAC could reverse this effect. Considering that MAPK/ERK and PI3K/AKT pathways exert great roles in thyroid oncogenesis and malignant progression [5], we also assessed the impact of DSF/Cu on these pathways. Our results indicated that DSF/Cu blocked their activities by decreasing the expression of ERK phosphorylation, total AKT and AKT phosphorylation, and NAC could reverse this effect. The above findings, taken together, indicate that DSF/Cu exerts its antitumor role in thyroid cancer through an ROS-dependent mechanism.

Considering that the reactivation of MAPK/ERK pathway is a major cause of the resistance to *BRAF* kinase inhibitors such as PLX4032 [16], we next determined the impact of DSF/Cu on the response of *BRAF*-mutated thyroid cancer cells to PLX4032. The results indicated that DSF/Cu enhanced antitumoral activity of PLX4032 in *BRAF^V600E^*-mutated thyroid cancer cells both in vitro and in vivo. Further experiments found that DSF/Cu sensitizes *BRAF*-mutated thyroid cancer cells to PLX4032 by suppressing the reactivation of *HER3* caused by PLX4032, thereby blocking MAPK and PI3K/AKT pathways, and NAC could reverse this effect. Altogether, our data indicate that DSF/Cu improves the response of *BRAF^V600E^*-mutated thyroid cancer cells to PLX4032 by ROS-dependently inhibiting *HER3* reactivation.

Our in vitro studies demonstrated that the combination of DSF/Cu and PLX4032 showed great antitumor activity. However, in vitro conditions are unable to sufficiently mimic the in vivo environment with hypoglycemia, hypoxia and other metabolic changes. Our data also demonstrated that DSF/Cu and PLX4032 significantly inhibited tumor growth in mouse models with no severe hepatorenal toxicity, indicating that DSF/Cu and PLX4032 can be used as a potential therapy for thyroid cancer. As an FDA-approved drug, disulfiram shows great safety in patients. Several clinical trials have shown that PLX4032 showed antitumor activity in patients [17]. Therefore, additional clinical trials will be needed to assess the clinical use of DSF/Cu and PLX4032 in thyroid cancer therapy and determine its safety and efficacy.

## 4. Materials and Methods

### 4.1. Cell Culture and Reagents

Human thyroid cancer cell lines 8505C, 8305C, IHH4, and BCPAP were provided by Haixia Guan (Guangdong Provincial People’s Hospital, Guangzhou, China). We cultured cells in RPMI 1640 medium (Gibco) with 10% fetal bovine serum (Gemini). In certain experiments, cells were treated with disulfiram (MCE), copper chloride (Sigma-Aldrich, St. Louis, MI, USA,), PLX4032 (Selleck) or N-acetylcysteine (MCE) at certain times and doses individually or in combination. We dissolved disulfiram and PLX4032 in dimethyl sulfoxide (DMSO), and dissolved copper chloride and N-acetylcysteine (NAC) in ddH2O. As a control, DMSO was used with the same volume.

### 4.2. Cell Proliferation Assay

We seeded cells (2500–4000/well) in 96-well culture plates. After an overnight culture, we cultivated cells with various concentrations of DSF/Cu or PLX4032, individually or in combination for different times. Then we carried out MTT assays to assess cell proliferation, as previously mentioned [61]. Half-maximal inhibitory concentration (IC50) was calculated using the Reed–Muench method. Three triplicates were done to determine each data point.

### 4.3. Colony Formation Assay

We seeded cells (2500–4000/well) in 12-well culture plates, then treated them in the specific experiments with drugs as indicated and kept them in culture for 8–10 days. Cells were fixed by methanol, followed by staining with 0.08% crystal violet. Next, cell colony number was counted by inverted microscopy. Three replicates were run for each experiment.

### 4.4. Detection of Cellular Reactive Oxygen Species (ROS)

We cultured cells with DSF and CuCl_2_ for 48 h. Cells were then resuspended in RPMI 1640 medium with 150 μM ROS probe 2′,7′-dichlorofluorescin diacetate (Sigma-Aldrich) and kept in the dark at 37 °C for 1.5 h. After cells were digested and resuspended, ROS was analyzed using flow cytometry. In some assays, we added 2 mM NAC to the cells to remove cellular ROS when cells were incubated with DSF/Cu. Each assay was carried out in triplicate.

### 4.5. Western Blot Analysis

After incubation under the indicated conditions, we used RIPA lysis buffer to lyse the cells. The same amount of protein lysate was then divided by 7–12% SDS-PAGE, followed by diverting onto PVDF membrane (Mannheim, Germany, Roche Diagnostics). The membrane was blocked at room temperature for 2.5 h using 5% bovine serum albumin. The membrane was then placed for 15 h at 4 °C with the primary antibodies. Immunoblotting signaling was visualized by the ECL detection system (Tanon) after the membrane immunoblotted using secondary antibodies (ZSGB-BIO). In Supplementary Table S1, we give the antibody information.

### 4.6. Cell Apoptosis

We incubated cells with DSF/Cu and PLX4032 for 48 h, singly or in combination. Cells were then collected, followed by staining with an apoptosis kit (4A Biotech) according to instructions. Finally, stained cells were analyzed via flow cytometry. Each assay was run in triplicate.

### 4.7. Animal Studies

We bought five-week-old nude female mice from GemPharmatech (Jiangsu, China). We subcutaneously injected 5 × 10^6^ 8305C cells into the axilla of these mice. After the tumors grew to 10–25 mm^3^ on average, we randomly divided these mice into 4 groups (5 mice/group). PLX4032 (50 mg/kg, gavage) and DSF/CuCl_2_ (DSF 50 mg/kg, CuCl_2_ 0.15 mg/kg, gavage) were carried out individually or in combination once a day. Mice were weighed and their tumors measured using a Vernier caliper every two days. According to the following formula, we calculated neoplasm volumes: width^2^ × length × 0.5. After 2-week treatment, we killed mice via cervical dislocation, then collected and weighed tumors. All animal experiments were ratified by Xi’an Jiaotong University Animal Center.

H&E and immunohistochemical (IHC) staining were administered as per our previous study [62,63].

### 4.8. Biosafety Evaluation

Mouse blood samples were centrifuged to get serum. The toxicity of viscera, including glutamic pyruvic transaminase (GPT/ALT), glutamic oxaloacetic transaminase (GOT/AST), urea nitrogen (BUN), and creatinine (CRE) were examined according to the kit’s protocols.

### 4.9. Statistical Analysis

We used two-way ANOVA and Student’s t-tests to compare the data using GraphPad Prism 8.0 software. We used CompuSyn Software to statistically analyze the synergistic effect of the two drugs with the Chou–Talalay method [35,37]. *p* < 0.05 was recognized as a statistically significant difference.

## 5. Conclusions

In summary, by a series of in vitro and in vivo studies, we demonstrated that DSF/Cu kills *BRAF^V600E^*-mutated thyroid cancer cells and improved their response to *BRAF* kinase inhibitors through relieving the feedback activation of MAPK/ERK and PI3K/AKT pathways in an ROS-dependent way. This study not only provides strong evidence to support the clinical use of DSF/Cu in cancer therapy but also suggests that the combination of DSF/Cu and *BRAF* kinase inhibitors may be a safe and effective strategy for the treatment of *BRAF^V600E^*-mutated thyroid cancers.

## Data Availability

All data generated or analyzed during this study are included in this article.

## Supplementary Materials

The following supporting information can be downloaded at https://www.mdpi.com/article/10.3390/ijms24043418/s1.

## References

[1] Kitahara C.M., Sosa J.A. (2016). The changing incidence of thyroid cancer. Nat. Rev. Endocrinol..

[2] Siegel R.L., Miller K.D., Fuchs H.E., Jemal A. (2022). Cancer statistics, 2022. CA Cancer J. Clin..

[3] Megwalu U.C., Moon P.K. (2022). Thyroid Cancer Incidence and Mortality Trends in the United States: 2000–2018. Thyroid.

[4] Lim H., Devesa S.S., Sosa J.A., Check D., Kitahara C.M. (2017). Trends in Thyroid Cancer Incidence and Mortality in the United States, 1974–2013. JAMA.

[5] Xing M. (2013). Molecular pathogenesis and mechanisms of thyroid cancer. Nat. Rev. Cancer.

[6] Xing M. (2008). Recent advances in molecular biology of thyroid cancer and their clinical implications. Otolaryngol. Clin. North Am..

[7] Xing M. (2005). *BRAF* mutation in thyroid cancer. Endocr. Relat. Cancer.

[8] Xing M. (2007). *BRAF* mutation in papillary thyroid cancer: Pathogenic role, molecular bases, and clinical implications. Endocr. Rev..

[9] Xing M., Alzahrani A.S., Carson K.A., Viola D., Elisei R., Bendlova B., Yip L., Mian C., Vianello F., Tuttle R.M. (2013). Association between *BRAF* V600E mutation and mortality in patients with papillary thyroid cancer. JAMA.

[10] Xing M., Alzahrani A.S., Carson K.A., Shong Y.K., Kim T.Y., Viola D., Elisei R., Bendlova B., Yip L., Mian C. (2015). Association between *BRAF* V600E mutation and recurrence of papillary thyroid cancer. J. Clin. Oncol..

[11] Guerra A., Fugazzola L., Marotta V., Cirillo M., Rossi S., Cirello V., Forno I., Moccia T., Budillon A., Vitale M. (2012). A high percentage of *BRAF^V600E^* alleles in papillary thyroid carcinoma predicts a poorer outcome. J. Clin. Endocrinol. Metab..

[12] Espinosa A.V., Porchia L., Ringel M.D. (2007). Targeting *BRAF* in thyroid cancer. Br. J. Cancer.

[13] Marotta V., Sciammarella C., Vitale M., Colao A., Faggiano A. (2015). The evolving field of kinase inhibitors in thyroid cancer. Crit. Rev. Oncol. Hematol..

[14] Marotta V., Chiofalo M.G., Di Gennaro F., Daponte A., Sandomenico F., Vallone P., Costigliola L., Botti G., Ionna F., Pezzullo L. (2021). Kinase-inhibitors for iodine-refractory differentiated thyroid cancer: Still far from a structured therapeutic algorithm. Crit. Rev. Oncol. Hematol..

[15] Joseph E.W., Pratilas C.A., Poulikakos P.I., Tadi M., Wang W., Taylor B.S., Halilovic E., Persaud Y., Xing F., Viale A. (2010). The RAF inhibitor PLX4032 inhibits ERK signaling and tumor cell proliferation in a V600E *BRAF*-selective manner. Proc. Natl. Acad. Sci. USA.

[16] Montero-Conde C., Ruiz-Llorente S., Dominguez J.M., Knauf J.A., Viale A., Sherman E.J., Ryder M., Ghossein R.A., Rosen N., Fagin J.A. (2013). Relief of feedback inhibition of *HER3* transcription by RAF and MEK inhibitors attenuates their antitumor effects in *BRAF*-mutant thyroid carcinomas. Cancer Discov..

[17] Brose M.S., Cabanillas M.E., Cohen E.E.W., Wirth L.J., Riehl T., Yue H., Sherman S.I., Sherman E.J. (2016). Vemurafenib in patients with *BRAF^V600E^*-positive metastatic or unresectable papillary thyroid cancer refractory to radioactive iodine: A non-randomised, multicentre, open-label, phase 2 trial. Lancet Oncol..

[18] Sala E., Mologni L., Truffa S., Gaetano C., Bollag G.E., Gambacorti-Passerini C. (2008). *BRAF* silencing by short hairpin RNA or chemical blockade by PLX4032 leads to different responses in melanoma and thyroid carcinoma cells. Mol. Cancer Res..

[19] Prahallad A., Sun C., Huang S., Di Nicolantonio F., Salazar R., Zecchin D., Beijersbergen R.L., Bardelli A., Bernards R. (2012). Unresponsiveness of colon cancer to *BRAF*(V600E) inhibition through feedback activation of EGFR. Nature.

[20] Hatzivassiliou G., Song K., Yen I., Brandhuber B.J., Anderson D.J., Alvarado R., Ludlam M.J., Stokoe D., Gloor S.L., Vigers G. (2010). RAF inhibitors prime wild-type RAF to activate the MAPK pathway and enhance growth. Nature.

[21] Poulikakos P.I., Zhang C., Bollag G., Shokat K.M., Rosen N. (2010). RAF inhibitors transactivate RAF dimers and ERK signalling in cells with wild-type *BRAF*. Nature.

[22] Subbiah V., Baik C., Kirkwood J.M. (2020). Clinical Development of *BRAF* plus MEK Inhibitor Combinations. Trends Cancer.

[23] Cvek B. (2012). Nonprofit drugs as the salvation of the world’s healthcare systems: The case of Antabuse (disulfiram). Drug Discov. Today.

[24] Shen M.L., Johnson K.L., Mays D.C., Lipsky J.J., Naylor S. (2001). Determination of in vivo adducts of disulfiram with mitochondrial aldehyde dehydrogenase. Biochem. Pharmacol..

[25] Iljin K., Ketola K., Vainio P., Halonen P., Kohonen P., Fey V., Grafstrom R.C., Perala M., Kallioniemi O. (2009). High-throughput cell-based screening of 4910 known drugs and drug-like small molecules identifies disulfiram as an inhibitor of prostate cancer cell growth. Clin. Cancer Res..

[26] Chen D., Cui Q.C., Yang H., Dou Q.P. (2006). Disulfiram, a clinically used anti-alcoholism drug and copper-binding agent, induces apoptotic cell death in breast cancer cultures and xenografts via inhibition of the proteasome activity. Cancer Res..

[27] Zha J., Chen F., Dong H., Shi P., Yao Y., Zhang Y., Li R., Wang S., Li P., Wang W. (2014). Disulfiram targeting lymphoid malignant cell lines via ROS-JNK activation as well as Nrf2 and NF-kB pathway inhibition. J. Transl. Med..

[28] Lovborg H., Oberg F., Rickardson L., Gullbo J., Nygren P., Larsson R. (2006). Inhibition of proteasome activity, nuclear factor-KappaB translocation and cell survival by the antialcoholism drug disulfiram. Int. J. Cancer.

[29] Yip N.C., Fombon I.S., Liu P., Brown S., Kannappan V., Armesilla A.L., Xu B., Cassidy J., Darling J.L., Wang W. (2011). Disulfiram modulated ROS-MAPK and NFkappaB pathways and targeted breast cancer cells with cancer stem cell-like properties. Br. J. Cancer.

[30] Allensworth J.L., Evans M.K., Bertucci F., Aldrich A.J., Festa R.A., Finetti P., Ueno N.T., Safi R., McDonnell D.P., Thiele D.J. (2015). Disulfiram (DSF) acts as a copper ionophore to induce copper-dependent oxidative stress and mediate anti-tumor efficacy in inflammatory breast cancer. Mol. Oncol..

[31] Skrott Z., Mistrik M., Andersen K.K., Friis S., Majera D., Gursky J., Ozdian T., Bartkova J., Turi Z., Moudry P. (2017). Alcohol-abuse drug disulfiram targets cancer via p97 segregase adaptor NPL4. Nature.

[32] Prasad S., Gupta S.C., Tyagi A.K. (2017). Reactive oxygen species (ROS) and cancer: Role of antioxidative nutraceuticals. Cancer Lett..

[33] Pelicano H., Carney D., Huang P. (2004). ROS stress in cancer cells and therapeutic implications. Drug Resist. Updat..

[34] Lopez-Lazaro M. (2007). Dual role of hydrogen peroxide in cancer: Possible relevance to cancer chemoprevention and therapy. Cancer Lett..

[35] Chou T.C. (2006). Theoretical basis, experimental design, and computerized simulation of synergism and antagonism in drug combination studies. Pharmacol. Rev..

[36] Chou T.C. (2010). Drug combination studies and their synergy quantification using the Chou-Talalay method. Cancer Res..

[37] Chou T.C., Talalay P. (1984). Quantitative analysis of dose-effect relationships: The combined effects of multiple drugs or enzyme inhibitors. Adv. Enzyme Regul..

[38] Cantwell-Dorris E.R., O’Leary J.J., Sheils O.M. (2011). *BRAF^V600E^*: Implications for carcinogenesis and molecular therapy. Mol. Cancer Ther..

[39] Roskoski R. (2021). Properties of FDA-approved small molecule protein kinase inhibitors: A 2021 update. Pharmacol. Res..

[40] Cabanillas M.E., Ryder M., Jimenez C. (2019). Targeted Therapy for Advanced Thyroid Cancer: Kinase Inhibitors and Beyond. Endocr. Rev..

[41] Subbiah V., Kreitman R.J., Wainberg Z.A., Cho J.Y., Schellens J.H.M., Soria J.C., Wen P.Y., Zielinski C.C., Cabanillas M.E., Boran A. (2022). Dabrafenib plus trametinib in patients with *BRAF* V600E-mutant anaplastic thyroid cancer: Updated analysis from the phase II ROAR basket study. Ann. Oncol..

[42] Chapman P.B., Hauschild A., Robert C., Haanen J.B., Ascierto P., Larkin J., Dummer R., Garbe C., Testori A., Maio M. (2011). Improved survival with vemurafenib in melanoma with *BRAF* V600E mutation. N. Engl. J. Med..

[43] Chapman P.B., Robert C., Larkin J., Haanen J.B., Ribas A., Hogg D., Hamid O., Ascierto P.A., Testori A., Lorigan P.C. (2017). Vemurafenib in patients with *BRAF*V600 mutation-positive metastatic melanoma: Final overall survival results of the randomized BRIM-3 study. Ann. Oncol..

[44] Sosman J.A., Kim K.B., Schuchter L., Gonzalez R., Pavlick A.C., Weber J.S., McArthur G.A., Hutson T.E., Moschos S.J., Flaherty K.T. (2012). Survival in *BRAF* V600-mutant advanced melanoma treated with vemurafenib. N. Engl. J. Med..

[45] Roskoski R. (2018). Targeting oncogenic Raf protein-serine/threonine kinases in human cancers. Pharmacol. Res..

[46] Hyman D.M., Puzanov I., Subbiah V., Faris J.E., Chau I., Blay J.Y., Wolf J., Raje N.S., Diamond E.L., Hollebecque A. (2015). Vemurafenib in Multiple Nonmelanoma Cancers with *BRAF* V600 Mutations. N. Engl. J. Med..

[47] Aida S., Sonobe Y., Tanimura H., Oikawa N., Yuhki M., Sakamoto H., Mizuno T. (2017). MITF suppression improves the sensitivity of melanoma cells to a *BRAF* inhibitor. Cancer Lett..

[48] Hu L., Zhang J., Tian M., Kang N., Xu G., Zhi J., Ruan X., Hou X., Zhang W., Yi J. (2022). Pharmacological inhibition of Ref-1 enhances the therapeutic sensitivity of papillary thyroid carcinoma to vemurafenib. Cell Death Dis..

[49] Wang J., Chen J., Miller D.D., Li W. (2014). Synergistic combination of novel tubulin inhibitor ABI-274 and vemurafenib overcome vemurafenib acquired resistance in *BRAF^V600E^* melanoma. Mol. Cancer Ther..

[50] Collins F.S. (2011). Mining for therapeutic gold. Nat. Rev. Drug Discov..

[51] Pantziarka P., Verbaanderd C., Huys I., Bouche G., Meheus L. (2021). Repurposing drugs in oncology: From candidate selection to clinical adoption. Semin. Cancer Biol..

[52] Ying H., Qin A., Cheng T.S., Pavlos N.J., Rea S., Dai K., Zheng M.H. (2015). Disulfiram attenuates osteoclast differentiation in vitro: A potential antiresorptive agent. PLoS ONE.

[53] Wang X., Yang S., Ye H., Chen J., Shi L., Feng L., Wang X., Zhang T., Chen R., Xiao W. (2022). Disulfiram Exerts Antiadipogenic, Anti-Inflammatory, and Antifibrotic Therapeutic Effects in an In Vitro Model of Graves’ Orbitopathy. Thyroid.

[54] Li H., Wang J., Wu C., Wang L., Chen Z.S., Cui W. (2020). The combination of disulfiram and copper for cancer treatment. Drug Discov. Today.

[55] Morrison B.W., Doudican N.A., Patel K.R., Orlow S.J. (2010). Disulfiram induces copper-dependent stimulation of reactive oxygen species and activation of the extrinsic apoptotic pathway in melanoma. Melanoma Res..

[56] Chiba T., Suzuki E., Yuki K., Zen Y., Oshima M., Miyagi S., Saraya A., Koide S., Motoyama T., Ogasawara S. (2014). Disulfiram eradicates tumor-initiating hepatocellular carcinoma cells in ROS-p38 MAPK pathway-dependent and -independent manners. PLoS ONE.

[57] Ohtaki S., Nakagawa H., Kimura S., Yamazaki I. (1981). Analyses of catalytic intermediates of hog thyroid peroxidase during its iodinating reaction. J. Biol. Chem..

[58] Massart C., Hoste C., Virion A., Ruf J., Dumont J.E., Van Sande J. (2011). Cell biology of H2O2 generation in the thyroid: Investigation of the control of dual oxidases (DUOX) activity in intact ex vivo thyroid tissue and cell lines. Mol. Cell. Endocrinol..

[59] Moreno J.C., Bikker H., Kempers M.J., van Trotsenburg A.S., Baas F., de Vijlder J.J., Vulsma T., Ris-Stalpers C. (2002). Inactivating mutations in the gene for thyroid oxidase 2 (THOX2) and congenital hypothyroidism. N. Engl. J. Med..

[60] Li Y., Chen F., Chen J., Chan S., He Y., Liu W., Zhang G. (2020). Disulfiram/Copper Induces Antitumor Activity against Both Nasopharyngeal Cancer Cells and Cancer-Associated Fibroblasts through ROS/MAPK and Ferroptosis Pathways. Cancers.

[61] Cui B., Yang Q., Guan H., Shi B., Hou P., Ji M. (2014). PRIMA-1, a mutant p53 reactivator, restores the sensitivity of TP53 mutant-type thyroid cancer cells to the histone methylation inhibitor 3-Deazaneplanocin A. J. Clin. Endocrinol. Metab..

[62] Wang S., Zhou X., Zeng Z., Sui M., Chen L., Feng C., Huang C., Yang Q., Ji M., Hou P. (2021). Atovaquone-HSA nano-drugs enhance the efficacy of PD-1 blockade immunotherapy by alleviating hypoxic tumor microenvironment. J. Nanobiotechnol..

[63] Wang N., Li Y., Wei J., Pu J., Liu R., Yang Q., Guan H., Shi B., Hou P., Ji M. (2019). TBX1 Functions as a Tumor Suppressor in Thyroid Cancer Through Inhibiting the Activities of the PI3K/AKT and MAPK/ERK Pathways. Thyroid.

