# Supplementary material for: Disulfiram/Cu Kills and Sensitizes BRAF-Mutant Thyroid Cancer Cells to BRAF Kinase Inhibitor by ROS-Dependently Relieving Feedback Activation of MAPK/ERK and PI3K/AKT Pathways
Source: Int J Mol Sci. 2023 Feb 8;24(4):3418. doi: 10.3390/ijms24043418 (PMC9968072; doi:10.3390/ijms24043418)
Supplement: Supplementary file 1 [file ijms-24-03418-s001.zip › ijms-2148087-supplementary.pdf]

Supplementary Materials

Table S1. The antibodies used in this study

Antibodies	Catalog#	Source
anti-β-Actin	A5441	Millipore-Sigma
anti-t-ERK	#4695	Cell Signalling Technology
anti-p-ERK	#4370	Cell Signalling Technology
anti-t-AKT	#4691	Cell Signalling Technology
anti-p-AKT Ser473	#4060	Cell Signalling Technology
anti-p-AKT Thr308	#13038	Cell Signalling Technology
anti-t-HER3	#4754	Cell Signalling Technology
anti-p-HER3	#2842	Cell Signalling Technology
anti-p-AKT Thr308 (IHC)	BS4009	Bioworld Technology
anti-t-HER3 (IHC)	2220243	ZENBIO

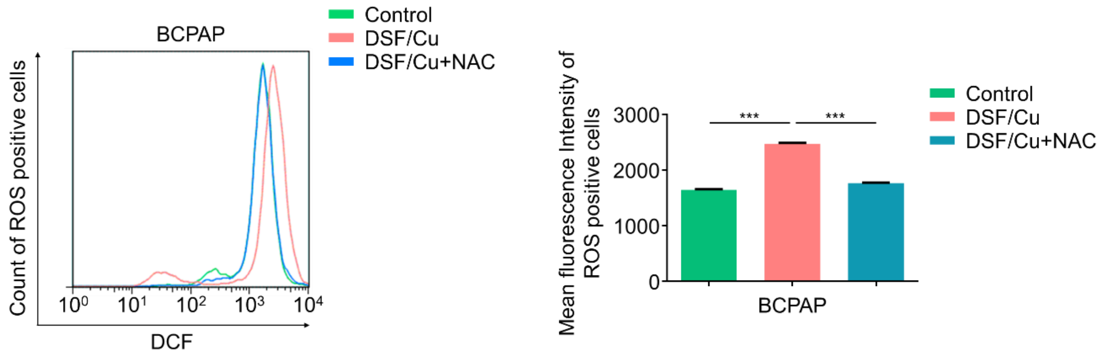

Figure S1. BCPAP cells were treated with 0.5 μM DSF/Cu alone or in combination with 2 mM NAC for 48 h, followed by a 1.5-h incubation with an ROS-sensitive fluorescent dye DCF-DA. The ROS-positive cells were measured by flow cytometer (left panel), and the mean fluorescence intensity of three independent experiments was then calculated by Student's t test (right panel). Data presented as means ± SD. *** $P < 0.001$.

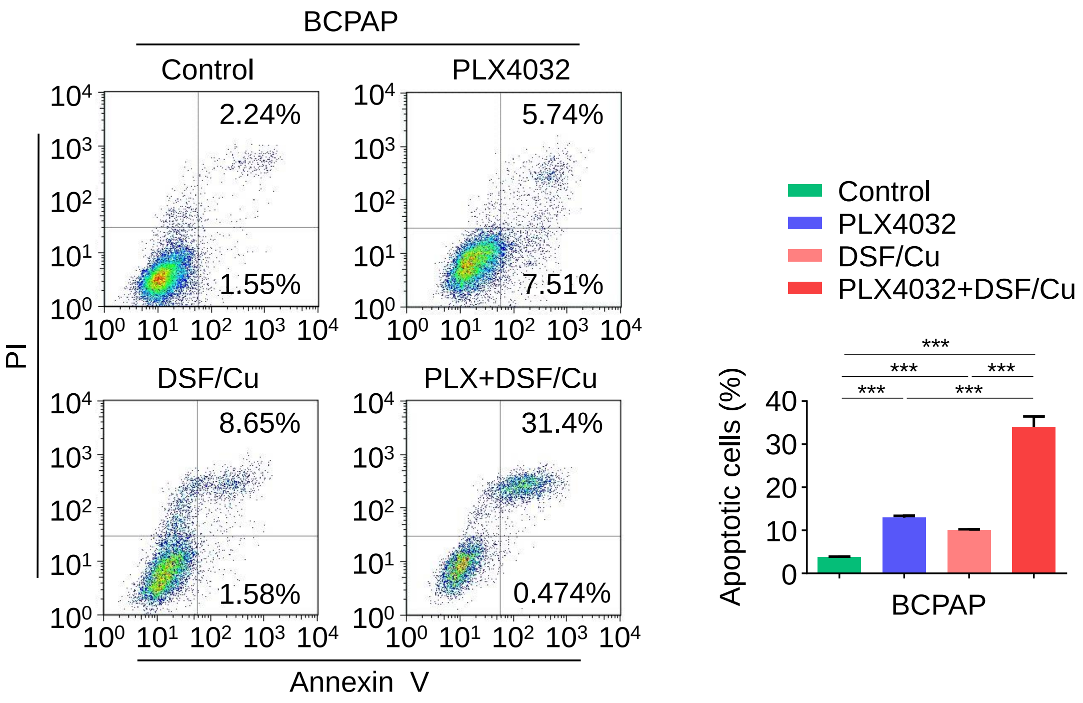

Figure S2. BCPAP cells were treated with DSF/Cu (500 nM: 500 nM) and PLX4032 (2 μM) , individually or in combination, for 48 h, and cell apoptosis was then measured by flow cytometry. Data presented as means ± SD. *** $P < 0.001$.

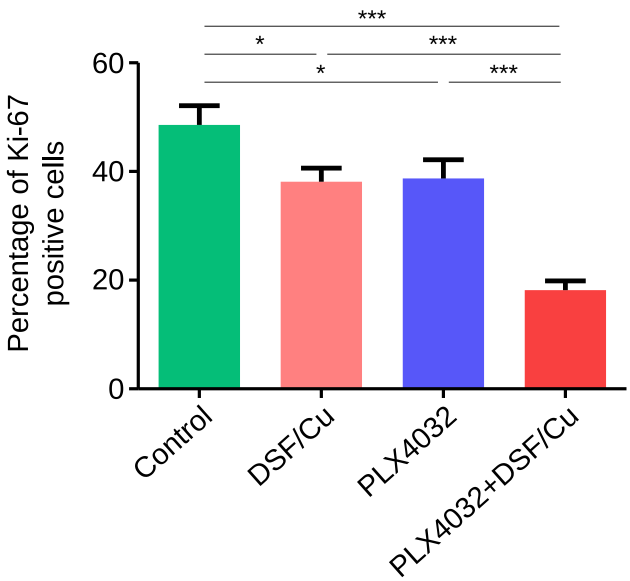

Figure S3. The percentage of Ki-67-positive cells was counted and compared among the indicated groups. Data presented as means ± SD. * $P < 0.05$; *** $P < 0.001$.
